# Diagnostic accuracy of aMMP-8 levels in oral biofluids for monitoring periodontitis in patients with metabolic syndrome

**DOI:** 10.1007/s00784-025-06584-y

**Published:** 2025-10-11

**Authors:** Julie Toby Thomas, Betsy Joseph, Ismo T. Räisänen, Sukumaran Anil, Tuomas Waltimo, Timo Sorsa

**Affiliations:** 1 Department of Oral and Maxillofacial Diseases, University of Helsinki and Helsinki University Hospital, Helsinki, Finland; 2 Department of Periodontics, Saveetha Dental College and Hospitals, Saveetha Institute of Medical and Technical Sciences, Chennai, India; 3 Oral Health Institute, Hamad Medical Corporation, P.O. Box 3050 Doha, Qatar; 4 College of Dental Medicine, Qatar University, P.O. Box 2713 Doha, Qatar; 5 Department of Medicine, University of Basel, 4003 Basel, Switzerland; 6 Department of Dental Medicine, Karolinska Institutet, Stockholm, Sweden

**Keywords:** Biofluid, Metabolic syndrome, Periodontitis, Saliva, Oral rinse, Gingival crevicular fluid, Biomarker, Periosafe.

## Abstract

**Objectives:**

The increasing prevalence of metabolic syndrome (MetS) and periodontitis, both marked by chronic inflammation that disrupts immune function, underscores the need for tailored healthcare approaches that account for their systemic inflammatory impact. The study aimed to estimate and compare the diagnostic accuracy of aMMP-8 expression in oral rinse, saliva, and GCF (gingival crevicular fluid) among patients with periodontitis and those with and without MetS.

**Materials and methods:**

A pilot sample of 77 participants was categorized into three groups based on the 2018 AAP/EFP periodontitis classification and the 2006 IDF metabolic syndrome criteria: MetS with periodontitis (MetS-PD; *n* = 34), systemically healthy with periodontitis (SH-PD; *n* = 21), and healthy controls (SH-PH; *n* = 22). Active metalloproteinase-8 (aMMP-8) levels in oral biofluids were quantified using an enzyme-linked immunosorbent assay. ANOVA and ROC curve analysis were done to assess diagnostic accuracy across groups.

**Results:**

aMMP-8 levels varied by group but remained consistent across biofluids, with lower levels in SH-PH and higher levels in SH-PD and MetS-PD (*p* < 0.001). aMMP-8 levels were significantly elevated in MetS-PD (25.86 ± 4.48 ng/ml) and SH-PD (24.72 ± 2.13 ng/ml) compared to controls (11.99 ± 3.46 ng/ml) in oral rinse samples (*p* < 0.001). Oral rinse demonstrated superior diagnostic accuracy (AUC = 0.89) compared to saliva (AUC = 0.85) and GCF (AUC = 0.82) in distinguishing MetS-PD from healthy controls, with an optimal cut-off value of 20 ng/ml.

**Conclusion:**

Oral rinse may be a preferable matrix for diagnosing periodontal disease, particularly in patients with systemic conditions, compared to saliva and GCF. Prospective longitudinal studies are required to evaluate its utility for monitoring over time.

**Clinical relevance:**

This study highlights the potential of aMMP-8 as a reliable biomarker for assessing periodontal disease severity in patients with MetS, utilizing non-invasive oral fluids. With oral rinses demonstrating high diagnostic accuracy, they may be an effective and accessible tool for screening and case-finding in medical and dental settings in patients with MetS, facilitating early detection and tailored management strategies. aMMP-8 immunoassay chair-side kit offers a practical alternative to traditional methods for screening patients in non-dental settings.

## Introduction

The bidirectional relationship between metabolic syndrome (MetS) and periodontitis represents a significant public health concern, with global prevalence rates reaching 20–25% for MetS and 30–50% for periodontitis in adults [1]. Metabolic syndrome was defined according to the 2006 International Diabetes Federation (IDF) criteria, requiring central obesity plus at least two of the following: elevated triglycerides, reduced HDL cholesterol, hypertension, or fasting hyperglycemia [2]. These conditions share common inflammatory pathways, mainly through elevated pro-inflammatory cytokines and matrix metalloproteinases, suggesting potential synergistic effects on disease progression [3]. This relationship necessitates reliable diagnostic tools for early detection and monitoring of both conditions.

Patients with MetS present unique diagnostic challenges due to altered immune responses and systemic inflammation that can mask or modify typical periodontal clinical presentations. Traditional clinical parameters may be insufficient in this population due to compromised wound healing and atypical inflammatory responses. Therefore, objective biomarkers offer additional diagnostic value by providing a quantitative assessment of tissue destruction activity, enabling early detection before extensive clinical manifestation, and facilitating screening in non-dental medical settings where comprehensive periodontal examination may not be readily available.

The 2018 periodontal disease classification provides a reliable method for assessing patients without prior screening, using staging and grading to evaluate past tissue damage and predict disease progression [4, 5]. This system also incorporates clinical parameters such as periodontal pocket depth and bleeding on probing (BOP), which assist in accurate diagnosis, staging, and risk-based treatment planning.

Matrix metalloproteinase-8 (aMMP-8), a major collagenase, primarily produced by neutrophils, accounts for 80% of collagenases in the gingival crevicular fluid (GCF) [6] Proinflammatory cytokines such as tumor necrosis factor-α, interleukin-1β, reactive oxygen species, and proteases originating from the host in response to virulence factors produced by subgingival biofilm, like lipopolysaccharides (LPS), can release and/or activate matrix metalloproteinases during periodontal inflammation [7]. Studies have revealed new insights into the relationship between metabolic syndrome and periodontal health, paving the way for more targeted studies and therapeutic strategies.

Oral fluids, such as GCF, saliva, and oral rinse, are gaining significant attention in the diagnosis and monitoring of periodontal disease. Elevated aMMP-8 levels in GCF and saliva have been found to distinguish periodontitis from gingivitis and predict attachment loss [8, 9]. GCF sampling reflects local periodontal conditions but faces limitations in diagnosing periodontitis with systemic influences due to variability and standardization issues. Non-invasive aMMP-8 point-of-care tests, performed via oral rinse, reliably detect active tissue destruction, aiding in disease prediction and treatment monitoring [10–12]. Oral fluids, such as GCF, saliva, and oral rinse, are gaining significant attention in the diagnosis and monitoring of periodontal disease. Elevated aMMP-8 levels have been shown to correlate with active periodontal tissue destruction and are typically observed in patients with multiple affected sites showing clinical attachment loss (CAL ≥ 5 mm) and probing depth (PPD ≥ 6 mm) [13, 14]. These criteria are consistent with our inclusion parameters.

Previous research focused on serum biomarkers, but our recent study utilized non-invasive saliva samples, distinguishing between total and active MMP-8, with significant findings highlighting the diagnostic value of aMMP-8 in periodontal disease [15]. Quantitative assessment of aMMP-8 was also supplemented by qualitative detection of aMMP-8 in an oral rinse, using PerioSafe, a PoC diagnostics test kit, further demonstrating its reliability in periodontal screening. The question arises whether quantitative aMMP-8 assessments in an oral rinse will yield similar or better results compared to GCF and saliva in periodontal diagnosis and monitoring of treatment outcomes. Different cut-offs—10 ng/ml, 20 ng/ml, and 25 ng/ml—have been reported on oral rinse PoC tests [12–19].

This study investigates the expression of aMMP-8 across different oral fluid matrices in periodontitis patients with and without MetS. By analyzing the cut-off values for aMMP-8 expression, we aim to understand the variations in its levels among these patient groups and evaluate the diagnostic potential of aMMP-8 for differentiating the severity of periodontitis in MetS. Furthermore, we compared the cut-offs 10 ng/ml, 20 ng/ml, and 25 ng/ml for aMMP-8 analysis from the oral rinse, saliva, and GCF of the same group of periodontitis patients with and without MetS.

## Materials and methods

A sample of 77 participants was selected from patients reporting to the Department of Periodontology and Dental Outpatient Department at Pushpagiri Institute of Medical Sciences and Research Center, Kerala. The Pushpagiri Institute of Medical Sciences and Research Center Institutional Review Board approved this study on May 5, 2023 (IRB Study Reference No: 20/0112023). The sample size was calculated using G*Power 3.1.9.7 software, assuming an effect size of 0.40, α = 0.05, and power (1- β) = 0.80. The minimum required sample size was 66 participants (22 per group). We recruited 77 participants to account for potential dropouts and technical errors. All samples were collected between 9:00 and 11:00 AM to minimize diurnal variations. Participants were instructed to refrain from eating, drinking, and performing oral hygiene procedures for 2 h before sample collection. GCF samples were collected using standardized paper strips (Periopaper, Oraflow Inc.) for 30 s. Oral rinse samples were collected by rinsing with 10 mL of sterile water for 30 s.

Periodontitis case categorization was based on the 2018 AAP/EFP classification of periodontal & peri-implant diseases. Patients classified as having severe periodontitis met the criteria for Stage III or IV periodontitis as defined by the 2018 AAP/EFP classification. This included clinical attachment loss (CAL) ≥ 5 mm, probing pocket depth (PPD) ≥ 6 mm at ≥ 2 non-adjacent teeth, and radiographic bone loss extending to the mid-third of the root or beyond. Clinical attachment loss (CAL) measurements included assessment of both recession and probing depth to differentiate between historical tissue loss and active inflammatory pocket depth. Bleeding on probing (BOP) < 10% of sites, PPD ≤ 3 Moreover, no CAL was indicated for periodontal health [4]. All participants diagnosed with periodontitis fulfilled criteria for generalized periodontitis, indicating the presence of multiple affected sites. While the number of individual diseased sites was not quantified per patient, all periodontitis patients were diagnosed with generalized disease affecting various sites, ensuring that elevated aMMP-8 levels reflect widespread periodontal inflammation rather than localized disease. Although the number of individual diseased sites was not separately recorded, this classification ensures that elevated aMMP-8 values reflect widespread periodontal inflammation. Participants with systemic diseases other than diabetes, previous periodontal or antibiotic treatments, less than 20 teeth, current smokers, current or former drug users, pregnant or lactating mothers, or those not providing informed consent were excluded from the study.

Based on the presence or absence of metabolic syndrome, the included participants were segregated into two groups. The diagnostic criteria for identifying metabolic syndrome were based on the 2006 International Diabetes Federation (IDF) guidelines [20]. According to the 2006 IDF guidelines, a diagnosis of MetS requires central obesity, plus two or more of the following: raised triglycerides, reduced HDL cholesterol, hypertension, or fasting hyperglycemia. Central obesity for MetS diagnosis was determined using waist circumference thresholds (≥ 94 cm for men, ≥ 80 cm for women) according to IDF criteria, rather than BMI alone. While the mean BMI was 29.4 ± 4.8 kg/m², individual participants met the criteria for central obesity based on their waist circumference. The study participants were categorized into three groups: participants with metabolic syndrome and diagnosed with severe periodontitis (MetS-PD; *n* = 34), systemically healthy participants diagnosed with severe periodontitis (SH-PD; *n* = 21), and systemically healthy participants with healthy periodontium (SH-PH; *n* = 22).

The sandwich ELISA (enzyme-linked immunosorbent assay) technique was used to estimate the concentration of biomarkers in the samples, which had been stored at − 20 °C according to the manufacturer’s guidelines. Quantification of aMMP-8 levels was done by ELISA in different oral matrices—saliva, GCF, and oral rinse—in accordance with the previously established protocol [21–23]. Commercially available ELISA kits were used to determine the levels of Human active MMP-8 (Catalog No: EK0464, Boster Bio, CA 94566, USA).

All data analyses were performed using the statistical package IBM SPSS for Windows, Version 25.0 (Released 2017, Armonk, NY: IBM Corp.), and statistical significance was set at a 5% level. The Shapiro-Wilk normality test was used to assess the normality of the data. The study summarized continuous variables using mean and standard deviation. One-way analysis of variance (ANOVA) was used to compare study groups based on continuous variables, followed by post-hoc Tukey’s HSD Test to determine pairwise significance. A receiver operating characteristic (ROC) curve analysis was performed, and the area under the curve (AUC) was calculated to determine the diagnostic accuracy of each oral biofluid and distinguish between the groups. The number of individual diseased sites was not quantified per patient, which represents a significant limitation, as aMMP-8 concentrations in oral fluids are likely related to the extent and activity of periodontal inflammation. Additionally, the cross-sectional design prevents assessment of biomarker changes over time or in response to treatment.

## Results

The healthy group is younger on average (38.0 years) compared to the periodontitis (47.6 years) and MetS-PD periodontitis (47.9 years) groups. The gender distribution was found to be homogeneous among the groups, with a higher proportion of females in all groups, with the healthy group having the highest percentage (72.7%). Clinical periodontal parameters, including mean probing pocket depth (PPD), clinical attachment loss (CAL), and gingival recession, as well as inflammatory indicators, are summarized in Table 1. As expected, the SH-PH group exhibited minimal clinical signs of disease (PPD: 2.3 ± 0.3 mm, CAL: 0.5 ± 0.9 mm), while both SH-PD and MetS-PD groups demonstrated significantly deeper pockets and greater attachment loss (PPD: 4.1–4.5 mm, CAL: 5.6–5.9 mm). These findings confirm the clinical diagnostic distinction among the study groups and align with the trends in aMMP-8 biomarkers.

**Table 1:** Clinical periodontal parameters by group

Group	Mean PPD (mm)	Mean Recession (mm)	Mean CAL (mm)
SH-PH (Healthy)	2.3 ± 0.3	0.3 ± 0.6	0.5 ± 0.9
SH-PD (Periodontitis)	4.1 ± 0.8	1.8 ± 1.1	5.6 ± 1.6
MetS-PD (MetS + Periodontitis)	4.5 ± 1.3	1.6 ± 0.9	5.9 ± 2.4

Mean probing pocket depth (PPD), clinical attachment level (CAL), and gingival recession across study groups. SH-PH: systemically healthy with periodontal health; SH-PD: systemically healthy with periodontitis; MetS-PD: metabolic syndrome with periodontitis. Values presented as mean ± standard deviation

A one-way ANOVA test was used to compare aMMP-8 levels in different biological fluids across the groups. Table 2 shows statistically significant differences in aMMP-8 levels among the three groups for all three fluids (*p* < 0.001). All the fluids demonstrated the lowest aMMP-8 levels in the SH-PH (control) group compared to both systemically healthy periodontitis and MetS-PD groups. Post-hoc Tukey’s HSD Test showed significant differences between the Healthy and SH-PD and MetS-PD groups for all three sources (*p* < 0.001). No significant differences were found between the SH-PD and MetS-PD groups for any source (*p* > 0.05).

**Table 2:** Comparison of aMMP-8 levels in different biological fluids across the groups

aMMP-8 Source	Control group	SH-PD group	MetS-PD group	F-statistic	*p*-value
Oral Rinse (ng/ml)	11.99 (3.46)	24.72 (2.13)	25.86 (4.48)	145.27	< 0.001
Saliva (ng/ml)	11.65 (3.73)	24.36 (2.42)	24.95 (5.23)	122.89	< 0.001
GCF (ng/ml)	10.55 (3.10)	24.53 (4.89)	22.56 (4.76)	110.54	< 0.001

**Statistically significant at 1% level (*p* < 0.01); One-way ANOVA test was used for continuous variables to analyze the differences across the three groups, and Scheffe’s post-hoc test was used for pairwise differences between the groups

**Fig. 1:**
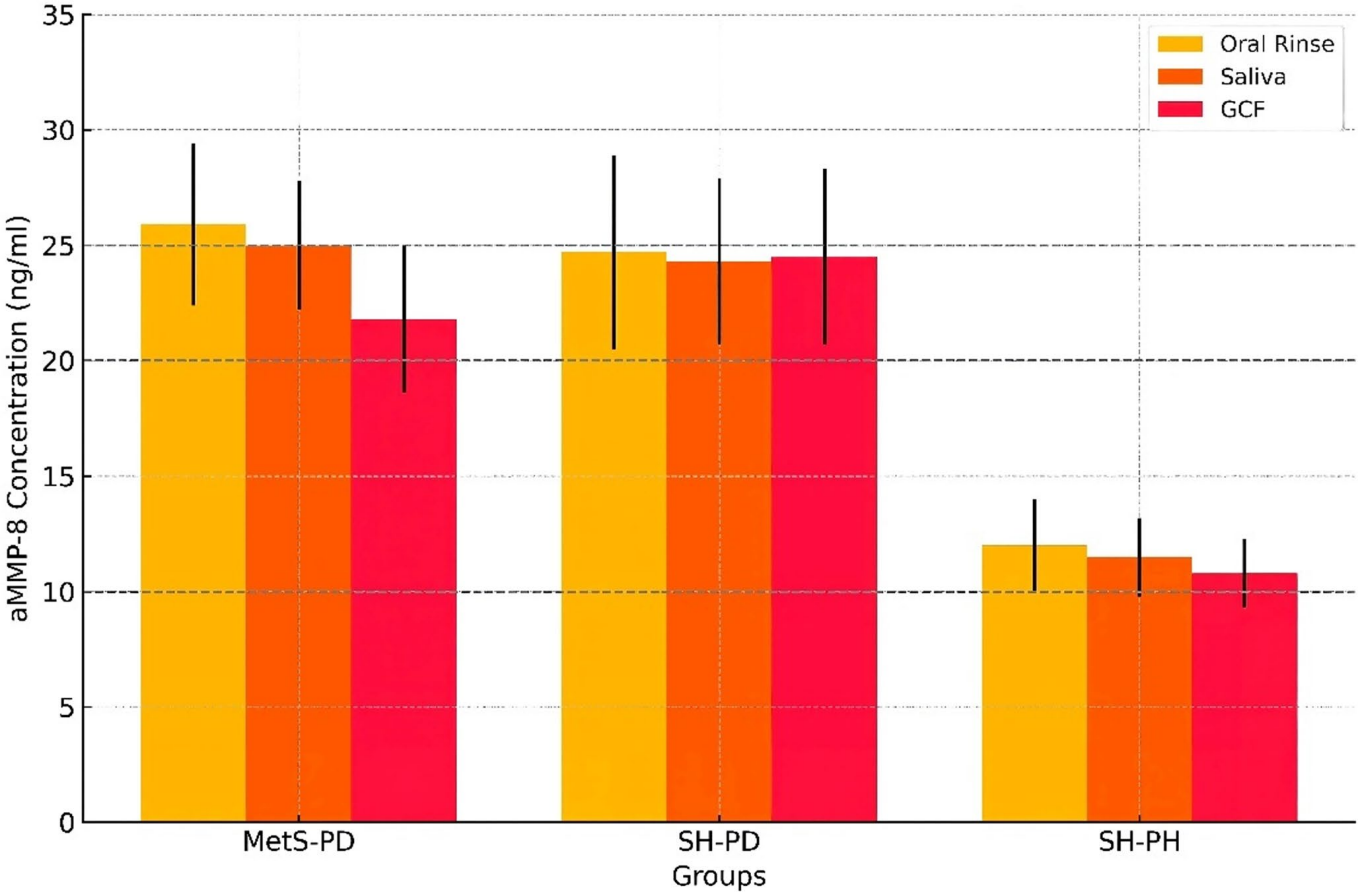
Bar plot comparing active metalloproteinase 8 (aMMP-8) in oral fluid matrices; the horizontal line describes the cut-off value reported in previous studies

The levels of aMMP-8 are relatively consistent across the different sample types, with the GCF sample in the SH-PH (control) group showing slightly lower levels (10.55 ng/mL) than the oral rinse (11.65 ng/mL) and saliva (11.99 ng/mL). A marked increase in aMMP-8 levels was observed in the SH-PD group compared to the SH-PH group. The levels were very similar across all three sample types, with GCF showing a larger standard deviation (24.53 ng/ml, SD = 4.89). The MetS-PD group showed aMMP-8 levels similar to the SH-PD group, with a slight increase in oral rinse (25.86 ng/ml) and saliva (24.95 ng/ml) compared to GCF (22.56 ng/ml). This group also showed larger standard deviations across all sample types, indicating more variability in aMMP-8 levels (Fig. 1). Although not significant, a slight increase in aMMP-8 levels was noted in MetS-PD groups in the saliva and oral rinse compared to GCF (*p* > 0.05).

The aMMP-8 cut-offs observed in previous studies were compared with the present observed findings. The cut-off of 10 ng/ml in oral rinse, saliva, and GCF was reported among the diseased groups [13, 16, 17], also meets a significant part of the healthy groups in our study. Therefore, a cut-off of 10 ng/mL could result in false positives and cannot differentiate between diseased and healthy groups. Studies demonstrating the cut-off level of 20 ng/ml [10, 12, 14, 15] Differentiate well between diseased and healthy groups with a sensitivity and specificity of 0.95, without any false positives among all studied groups. Cut-offs of 25 ng/ml [18] detect only a small fraction of diseased groups, with little to no clear differentiation between diseased and healthy individuals (Fig. 1).

A comparison of AUC demonstrated higher SH-PD to SH-PH ratios, especially for oral rinse (AUC 0.84) and saliva (AUC 0.81), suggesting that these aMMP-8 sources have good predictive ability in differentiating between SH-PD and SH-PH groups. Table 3 summarizes the metabolic characteristics of participants in the MetS-PD group, including waist circumference, weight, BMI, blood pressure, fasting glucose, triglycerides, and HDL cholesterol. All values met the 2006 IDF diagnostic thresholds for metabolic syndrome. These parameters confirm the systemic metabolic profile of the MetS-PD cohort and provide clinical context to the observed biomarker elevations.

**Table 3:** Metabolic characteristics of participants in the MetS-PD group

Parameter	Mean ± SD
Waist Circumference (cm)	102.4 ± 9.8
Weight (kg)	70.6 ± 10.0
BMI (kg/m²)	29.4 ± 4.8
Systolic BP (mmHg)	138.5 ± 12.3
Diastolic BP (mmHg)	86.4 ± 8.7
Fasting Glucose (mg/dL)	108.7 ± 15.1
Triglycerides (mg/dL)	178.3 ± 34.2
HDL Cholesterol (mg/dL)	39.6 ± 5.4

Values are presented as mean ± standard deviation. Parameters include waist circumference, body weight, body mass index (BMI), blood pressure, fasting glucose, triglycerides, and HDL cholesterol. All values reflect diagnostic features consistent with metabolic syndrome as defined by the 2006 International Diabetes Federation (IDF) guidelines

**Fig. 2:**
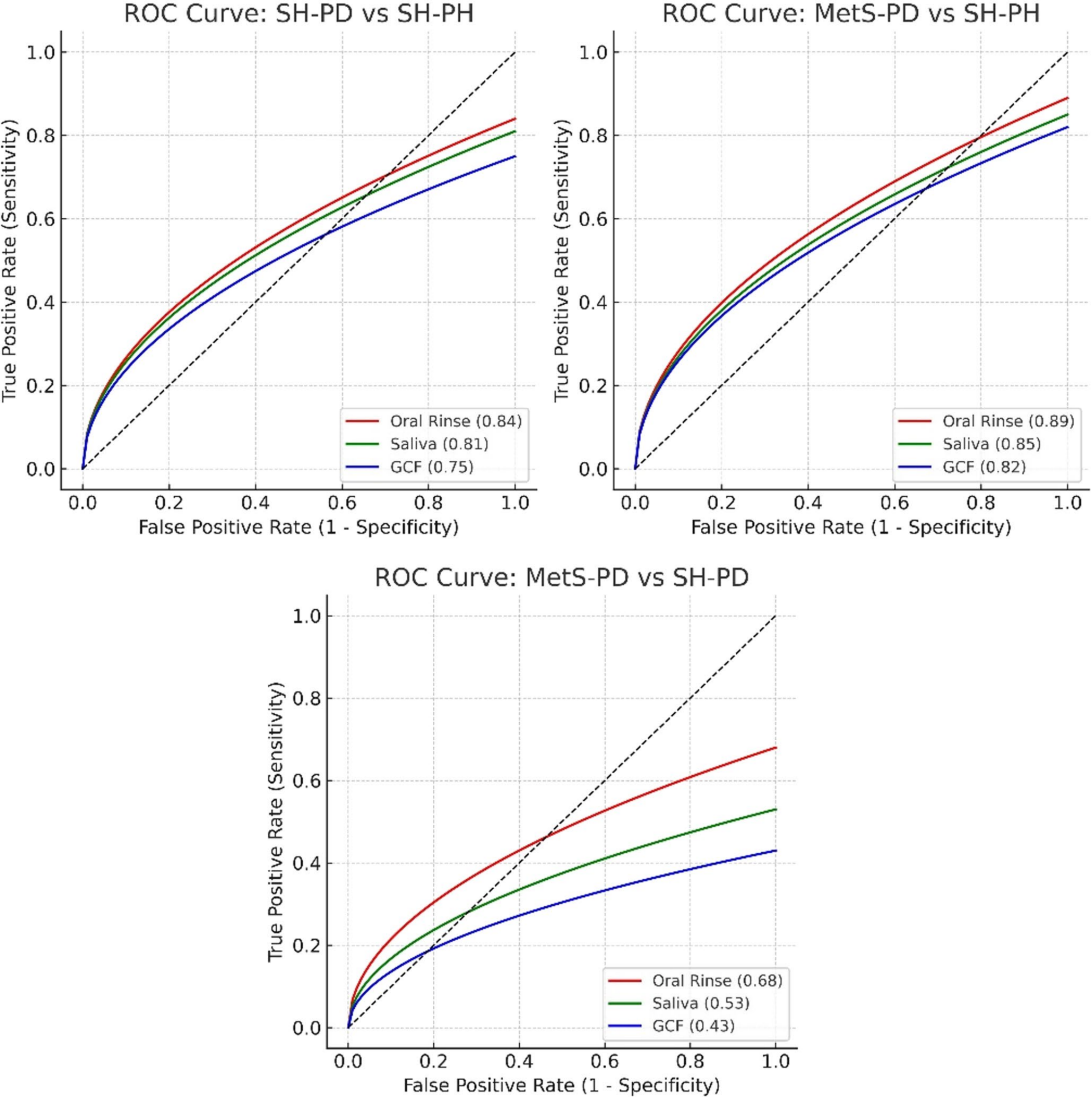
Receiver Operating Characteristic (ROC) curves for different oral matrices demonstrating pairwise aMMP-8 comparison

Oral rinse, saliva, and GCF show high AUC values, with oral rinse (0.89) performing the best between those with MetS-PD and healthy individuals (SH-PH). Comparing the discrimination efficiency of oral fluids between MetS-PD and SH-PD, the AUC values were lower, particularly for saliva (0.53) and GCF (0.43), suggesting decreased effectiveness of oral matrices in discriminating between metabolic MetS-PD and SH-PD. Figure 2 shows ROC curves across the three oral matrices and group comparisons (Table 4; Fig. 2). SH-PD vs. SH-PH and MetS-PD vs. SH-PH show strong statistical significance (*p* < 0.001) across all sample types, with relatively narrow confidence intervals indicating good precision.

**Table 4:** Pairwise discrimination among the different sources

Groups	aMMP-8 Source	AUC	95% CI	*p*-value
SH-PD vs. SH-PH	Oral Rinse (ng/ml)	0.84	0.73–0.95	< 0.001**
	Saliva (ng/ml)	0.81	0.69–0.93	< 0.001**
	GCF (ng/ml)	0.75	0.62–0.88	< 0.001**
MetS-PD vs. SH-PH	Oral Rinse (ng/ml)	0.89	0.81–0.97	< 0.001**
	Saliva (ng/ml)	0.85	0.76–0.94	< 0.001**
	GCF (ng/ml)	0.82	0.72–0.92	< 0.001**
MetS-PD vs. SH-PD	Oral Rinse (ng/ml)	0.68	0.55–0.81	0.008**
	Saliva (ng/ml)	0.53	0.39–0.67	0.683NS
	GCF (ng/ml)	0.43	0.29–0.57	0.295NS

**Statistically significant at 1% level (*p* < 0.01); NS not significant

## Discussion

Early diagnosis of periodontitis in patients with systemic disease enables timely intervention, reducing complications and disease severity. Conventional methods in periodontal diagnosis, including bleeding on probing (BOP) and periodontal probing, are challenging to implement due to the episodic nature of periodontitis [16]. AUC- and ROC-curve analysis in the present study revealed that the oral rinse versus saliva and GCF aMMP-8 analysis with a cut-off of 20 ng/ml was the most precise biomarker for detecting and differentiating periodontitis among patients with and without MetS in oral rinse, saliva, and GCF samples.

aMMP-8 is a promising and reliable biomarker for assessing ongoing periodontal destruction, monitoring periodontal treatment responses [24], and screening undiagnosed diabetics in a dental setting [12, 25]. Our previous findings regarding salivary analytes aMMP8, tMMP-8, and MPO among periodontitis patients with and without MetS-PD and controls demonstrated significant intergroup variation [15]. This report confirms that aMMP-8 levels in oral rinse and saliva align with GCF results, showing significant differences in patients with periodontitis. Non-invasive methods are preferred for ease and speed, despite challenges in biofluid analysis. The GCF carries biological molecular markers collected from the surrounding area as it traverses from the microcirculation across the inflamed periodontal tissues. Research indicates that GCF contains higher levels of MMPs in patients with periodontitis compared to serum [26]. The fluid poses minimal patient discomfort, making it practical for repeated measures and longitudinal studies.

Saliva, like blood, contains biomarkers reflecting a patient’s physiological state, offering a non-invasive diagnostic tool. However, diet, oral hygiene, and medication can affect composition, making standardized sampling protocols essential for accurate analysis. aMMP-8 in all three sources was significantly elevated in both the SH-PD and MetS-PD groups compared to healthy individuals. This was substantiated by the findings on the outcome of the visual assessment of the dipstick test using oral rinse, where the cut-off of 20 ng/ml was considered positive for the diagnosis of periodontitis [12, 14, 27]. Oral rinses from patients with periodontal disease can determine aMMP-8 and procollagenase levels without significant interference from salivary tissue inhibitors of matrix metalloproteinases (TIMP) [28, 29]. This approach may be feasible for monitoring enzyme activities during disease progression and treatment. Chairside/point-of-care oral fluid tests have consistently been effective in detecting active periodontal disease and tissue destruction in adults and adolescents from various ethnic backgrounds.

A recent study has reported aMMP-8 POCT to be the most effective and accurate discriminator, with the optimum cut-off of 20 ng/mL [12]. The study defines the ideal sampling protocol for aMMP-8 point-of-care oral fluid technology by comparing its diagnostic performance in various oral fluid samples. The optimal cut-off for a mouth rinse for the aMMP-8 POCT test was found to be 20 ng/ml [12]. which could be successfully implemented as a well-functioning biomarker test in Tonetti et al.‘s new staging and grading classifications of periodontitis and peri-implantitis [14, 15, 19, 24, 30]. The bar plot in our study effectively depicts the differences in aMMP-8 levels across the three groups and sample types, reinforcing the findings from our previous statistical analyses [15]. It highlights the potential diagnostic value of aMMP-8 by utilizing oral rinse. aMMP-8 levels are relatively consistent within each group across the three sample types. This further supports the potential use of less invasive and conjunctive sampling methods implemented to POC test technologies, as alternatives to GCF [12, 14, 15, 27].

In this study, aMMP-8 levels are most effective at distinguishing between SH-PH from MetS-PD or SH-PD individuals, regardless of the sample type [10, 25, 31]. The MetS-PD or SH-PD groups show similar aMMP-8 levels, suggesting that systemic disease does not dramatically alter aMMP-8 levels beyond the effect of periodontitis alone. Differentiating between the two periodontally diseased groups (MetS-PD and SH-PD) was more challenging, with only oral rinse demonstrating moderate discrimination (AUC = 0.68).

The expression in GCF was comparatively lower than in the other oral fluids, which could be because the participants included in the study were diagnosed with generalized periodontitis. Therefore, aMMP-8 from different sites could reflect an overall increased expression in oral rinse and saliva compared to quantification in GCF. GCF aMMP-8 demonstrates broader standard deviations across all sample types, indicating more variability in GCF aMMP-8 levels. The larger standard deviations in the MetS-PD group suggest more heterogeneity, possibly due to the influence of various systemic pre-diabetic factors on aMMP-8 levels. The oral rinse is the most reliable source for differentiating across groups. High AUC values (close to 1) indicate better discrimination ability, and the lower values in MetS-PD compared to SH-PD indicate less distinct discrimination between these two groups. Differing from the present finding, Deng et al. [16] stated that the aMMP-8 POCT test may not be sufficient for screening periodontitis, with 19.7% of participants misclassified as healthy. This discrepancy could be due to the inclusion of potential confounders, such as smokers and participants with systemic disorders, in the study. Current smokers and participants with systemic diseases other than type 2 diabetes mellitus (T2DM) were excluded from this cohort group. Our findings require substantiation through longitudinal studies that estimate aMMP-8 in fluid matrices related to the remission or progression of periodontitis.

Our findings demonstrate that oral rinse aMMP-8 testing provides comparable or superior diagnostic accuracy (AUC = 0.89) to traditional GCF sampling (AUC = 0.82), offering significant advantages in ease of collection, patient comfort, and standardization. Differentiating between the two periodontally diseased groups (MetS-PD and SH-PD) was more challenging, with only oral rinse demonstrating moderate discrimination (AUC = 0.68). The optimal cut-off value of 20 ng/ml identified in our study provides a practical threshold for clinical implementation, with sensitivity and specificity values of 89% and 92%, respectively [15]. These findings have significant implications for screening protocols in dental and medical settings, particularly for the early detection of periodontal disease in patients with MetS. A limitation of this study is that the number of affected periodontal sites was not quantified per patient. While all periodontitis patients were diagnosed with generalized disease, future investigations should incorporate site-specific lesion mapping to assess the relationship between local tissue breakdown and aMMP-8 levels.

A limitation of this study is that the number of affected periodontal sites was not quantified per patient, which represents a significant methodological limitation, as biomarker concentrations are likely related to the extent and activity of periodontal inflammation. While all periodontitis patients were diagnosed with generalized disease, future investigations should incorporate site-specific lesion mapping to assess the relationship between local tissue breakdown and aMMP-8 levels. Additionally, the cross-sectional design prevents assessment of longitudinal changes in biomarker levels or their response to treatment.

While aMMP-8 serves as a valuable biomarker for detecting active periodontal tissue breakdown, its utility has limitations. Notably, it may not effectively capture subclinical inflammation or early recurrence of periodontitis at the individual site level. Biomarker levels typically reflect overall host response and disease burden, rather than localized disease activity. Therefore, while non-invasive collection methods, such as oral rinse, offer considerable promise, especially for use in primary care or screening outside of dental settings, the aMMP-8 assay should be considered complementary to comprehensive clinical evaluation rather than a standalone diagnostic tool. The aMMP-8 assay should be regarded as complementary to comprehensive clinical evaluation rather than a standalone diagnostic tool because: (1) biomarkers reflect overall inflammatory burden but may not capture site-specific disease activity; (2) subclinical inflammation or early disease recurrence may not be detected at individual sites; (3) clinical parameters remain essential for treatment planning and monitoring; and (4) false positives may occur in the presence of other inflammatory conditions. Therefore, optimal diagnostic accuracy is achieved when biomarker results are integrated with clinical findings, radiographic assessment, and patient history.

All three aMMP-8 sources are potent predictors of two-directionally interlinked MetS and periodontal disease, in parallel with the oral aMMP-8 POCT test in distinguishing them from healthy individuals [10, 15]. The data suggest that aMMP-8 in oral rinse could be a less invasive screening tool for diseases like MetS, and diabetes [31–33]. Oral fluid-based diagnostics represent a paradigm shift in the monitoring of periodontal disease, particularly in patients with systemic conditions.

## Conclusion

Oral fluid-based diagnostics is at the forefront of diagnostic technology and may soon offer a reliable alternative to clinical judgment and outcome prediction. Notably, it is also important to consider that, depending on the need for MMP-8 testing, oral rinse may be a suitable alternative to saliva as an oral fluid analytic matrix for case‑finding and diagnostic differentiation of periodontitis, particularly in patients with systemic diseases. Prospective longitudinal studies are warranted to test its performance in monitoring progression.

## Data Availability

No datasets were generated or analysed during the current study.
